# The brain at war: effects of stress on brain structure in soldiers deployed to a war zone

**DOI:** 10.1038/s41398-021-01356-0

**Published:** 2021-04-26

**Authors:** Simone Kühn, Oisin Butler, Gerd Willmund, Ulrich Wesemann, Peter Zimmermann, Jürgen Gallinat

**Affiliations:** 1 Lise Meitner Group for Environmental Neuroscience, Max Planck Institute for Human Development, Berlin, Germany; 2 University Medical Center Hamburg-Eppendorf (UKE), Department of Psychiatry and Psychotherapy, Martinistrasse 52, 20246 Hamburg, Germany; 3 Center for Military Mental Health, Military Hospital Berlin, Scharnhorststraße 13, 10115 Berlin, Germany

**Keywords:** Pathogenesis, Neuroscience

## Abstract

In search of the neural basis of severe trauma exposure and post-traumatic stress disorder (PTSD), a multitude of cross-sectional studies have been conducted, most of them pointing at structural deficits in the hippocampus and medial prefrontal cortex including the anterior cingulate cortex (ACC) and ventromedial prefrontal cortex (vmPFC). Since cross-sectional studies are silent to causality, the core question remains: which brain structural alterations constitute a risk factor for disease and therewith precede the stressor, and which brain regions may undergo alterations as a consequence of exposure to the stressor. We assessed 121 soldiers before and after deployment to regions of war and 40 soldiers as controls, who were not deployed. Analysis using voxel-based morphometry revealed volumetric reductions in the ACC, vmPFC (region of interest analysis, effect does not survive conservative multiple test correction) and in bilateral thalamus (whole-brain analysis) in the deployment group. Remarkably, the ACC and vmPFC volume decrease was not limited to the period of deployment, but continued over the following 6 months after deployment. Volumetric reductions did not correlate with increases in PTSD symptoms. The volume decreases in medial prefrontal cortex and thalamus seem to be driven by trauma exposure rather than a vulnerability factor for PTSD. However, data indicate that the volume decrease in medial prefrontal cortex surpasses the time period of deployment. This may hint at an initiated pathobiological process below a symptom threshold, potentially paving the way to future mental health problems.

## Introduction

Stress is an ubiquitous phenomenon in our daily lives and its negative impact on mental and physical health has long been recognized^1^. However, effects of extremely stressful life events such as the experience of acts of terrorism, natural disaster or military combat are difficult to study, since these events are rare and less predictable. Importantly, individuals respond very differently to exposure to traumatic events, with some showing resilience^2^ and others developing psychiatric diseases such as post-traumatic stress disorder (PTSD) or depression^3^. In search of the neural basis of trauma exposure and/or PTSD a multitude of cross-sectional studies have been conducted, most of them pointing at brain structural deficits in hippocampus (HC) and medial prefrontal cortex including anterior cingulate cortex (ACC) and ventromedial prefrontal cortex (vmPFC) in patients in comparison to trauma-exposed controls^4,5^. However, cross-sectional studies as well as prospective studies that commonly only start after traumatic events^6,7^ do not provide any information about causation or its direction. A still unanswered question is how to best determine whether brain structural alterations are a risk factor for disease and therewith precede trauma and/or onset of the disorder, or whether the alterations are a consequence of exposure to trauma and/or the disease. In order to solve this question, monozygotic (MZ) twin studies have been conducted in which twins were identified of whom one received a diagnosis of PTSD (discordant for PTSD) and other twin pairs who were discordant for trauma exposure. Since MZ twin pairs share the same genes and most of the environment during upbringing, this study design can be used to identify whether structural alterations are a risk factor for, or a consequence of disease. A twin study by Gilbertson and colleagues revealed that smaller hippocampal volumes constitute a vulnerability factor rather than a product of trauma exposure^8^.

Another study by the same group reported a diagnosis × trauma exposure interaction in which the combat-exposed twins with PTSD had lower gray matter density in rostral ACC than their own, combat-unexposed co-twins as well as other combat-exposed twins without PTSD, supporting the inference that pregenual ACC gray matter reduction is an acquired sign of PTSD^9^. However, these reported results do not show what structural alteration are related to trauma exposure per se, and rely on a small sample of veterans with chronic PTSD.

In order to unravel the brain structural effects of trauma as such, prospective studies are needed with assessments before and after exposure to trauma^10^ in individuals who do not develop PTSD as a result of those experiences. In a first study of this kind by van Wingen and colleagues^11–13^ no structural gray matter changes were observed, however the sample size was limited (*N* = 33 combat-exposed soldiers, *N* = 26 controls); in another study the observed structural changes were not compared to a control group (*N* = 37 earthquake survivors)^14^. To address this key question, we acquired data from a large sample of active service members from the German military (referred to as “soldiers” in the following) who underwent brain imaging before and after deployment to a combat zone, which is typically associated with stressful/traumatic experiences. Additionally, we recruited a control group of soldiers who were not deployed during the respective period in time.

## Methods

### Participants

Soldiers were recruited from the German military by contact to military units scheduled for deployment. The combat group of whom we obtained magnetic resonance imaging (MRI) data pre and post deployment consisted of 121 soldiers (9 female) without known psychiatric or neurological disorders or any severe medical condition. The soldiers were deployed either to Afghanistan, Mali, Kosovo or Iraq (between 2012–2017) for 4.33 months (131 days) on average (SD = 38.95). They were exposed to typical war-zone stressors according to the Combat Experiences Scale of the US Armed Forces Mental Health Advisory Team (CES).

A control group of 40 soldiers (5 female), who were not deployed in the respective time window, was also assessed. From the combat group 67 of the 121, from the control group 17 of the 40 were previously deployed (Table 1).

**Table 1:** Descriptive information on study sample.

	Combat group (*n* = 121)		Control group (*n* = 40)		Statistics	
	Baseline	Follow-up I	Baseline	Follow-up I	Baseline^a^	Group × time^b^
	Mean (SD)	Mean (SD)	Mean (SD)	Mean (SD)	*t*- and *p*-value	*F*- and *p*-value
Age (years)	32.6 (8.5)		29.7 (6.3)		2.02, 0.045*	
Sex (m/f)	112/9		35/5		0.97, 0.325^c^	
Lifetime days of deployment	188.6 (285.1)		106.5 (184.6)		1.71, 0.090	
No. of subjects with previous deployments (yes/no)	67/54		17/23		2.00, 0.158^c^	
Digit symbol score	55.7 (9.8)		56.7 (8.9)		−0.60, 0.550	
*Questionnaires:*						
PTSD symptomatology (PDS Total)	2.49 (3.43)	2.53 (3.86)	3.44 (4.88)	2.52 (4.11)	−0.75, 0.456	1.33, 0.251
Anxiety (STAI State)	31.80 (6.59)	32.50 (7.26)	33.50 (5.73)	33.90 (6.21)	−1.42, 0.157	0.05, 0.847
Anxiety Sensitivity (ASI)	14.24 (7.60)	13.67 (7.50)	13.60 (7.46)	12.98 (6.49)	0.465, 0.643	0.001, 0.977
Depressivity (BDI)	2.60 (3.16)	3.36 (4.21)	3.05 (3.69)	2.53 (3.96)	−0.776, 0.451	5.25, 0.023*
Rumination (RSQ)	33.95 (7.73)	33.55 (8.82)	33.48 (8.96)	33.55 (8.76)	0.32, 0.747	0.41, 0.525
Alcohol drinking (AUDIT)	6.63 (4.28)	6.06 (4.13)	5.55 (4.01)	5.73 (4.01)	1.40, 0.165	1.48, 0.226

^a^independent *t*-test.

^b^Group × time resulting from a repeated measures analysis of variance.

^c^Pearson *χ*^2^-test.

*p < 0.05.

The number of participants was based on a sample size calculation for a repeated-measures ANOVA with two time points and two groups assuming an effect size of *f* = 0.3, and a power of 0.95. However, since we also wanted to be able to conduct within group comparisons in the deployment group, we decided to split the 160 up into 120 soldiers with and 40 without deployment.

The study was in accordance with the declaration of Helsinki and approved by the local ethics committee of Charité University Clinic. All participants provided written informed consent after receiving a written and oral description of the study.

All soldiers in the present sample had baseline PTSD-scores (Post-traumatic Diagnostic Scale (PDS)^15^) that were below the cut-off for clinical diagnosis (cut-off=28, suggested by the authors of PDS^16^, as well as with a more conservative cut-off = 24, suggested based on a German sample^17^).

Soldiers in the combat group were scanned before (Baseline) and after deployment (Follow-up I) with a mean interval of 216 days (SD = 68 days), soldiers in the control group were assessed with a mean interval of 214 days (SD = 85 days), with no significant difference between groups (*t*(158)=0.13, *p* = 0.90). Soldiers in the combat group were scanned a third time (Follow-up II, *n* = 92) on average 189 days (SD = 107 days) after Follow-up I.

### Scanning procedure

Structural images were collected on a Siemens Tim Trio 3 T scanner and a standard 12-channel head coil. The structural T1-weighted images were obtained using a magnetization prepared gradient-echo sequence (MPRAGE) (repetition time = 2500 ms; echo time = 4.77 ms; TI = 1100 ms, acquisition matrix = 256 × 256 × 176, flip angle = 7˚; 1 × 1 × 1 mm voxel size).

### Voxel-based morphometry (VBM)

We obtained gray matter volume estimates using CAT12 (v1278) running on SPM12 and Matlab R2016b. Longitudinal processing was performed using default parameters (http://www.neuro.uni-jena.de/cat12/CAT12-Manual.pdf). CAT12 automatically performs intra-subject realignment, bias correction, segmentation, and normalization (normalization is estimated for the mean image of all time points and then applied to all images). The extracted gray and white matter maps were smoothed using a 6 mm FWHM kernel. Images were visually inspected for artefacts prior to processing. A statistical quality control based on inter-subject homogeneity after segmentation was conducted. Segmented images were then visually inspected again.

### Questionnaires

To assess psychological symptoms, soldiers completed the following self-report questionnaires: Posttraumatic Diagnostic Scale (PDS), State Trait Anxiety Inventory state (STAI), Anxiety Sensitivity Inventory (ASI), Beck Depression Inventory (BDI-II), Response Style Questionnaire (RSQ), and the Alcohol Use Disorder Identification Test (AUDIT) in its German version. A detailed description of the instruments can be found in the Supplementary Material.

### Statistical analysis

We conducted group × time analyses on the self-report questionnaires, while controlling for age, since we observed a significant group difference in age (Table 1). In the absence of an expected group x time interaction we used Bayesian statistics as implemented in JASP. For BDI we used a non-parametric robust repeated measures ANOVA with a 10% trimmed mean^18^.

For region of interest (ROI) analyses, we extracted mean gray matter volume from clusters, which were the result of a meta-analysis on structural differences between patients with a PTSD diagnosis vs. trauma-exposed controls^4^, namely ACC (0, 40, 21), vmPFC (0, 49, 6) and left HC (−30, −14, −14) using the tool MarsBaR (http://marsbar.sourceforge.net/^19^). Additionally, we used an anatomical amygdala mask based on the Automatic Labeling Atlas^20^.

The whole-brain analysis was conducted on GM and WM segmentations. Since we were focusing on within-subject changes, we did not control for covariates such as sex and total intracranial volume (TIV), but for age (at each respective timepoint of MRI acquisition), because we found a significant group difference at Baseline (Table 1). We ran a group × time interaction analysis to identify regions where with group differences in differences over time. The resulting statistical maps were thresholded with *p* < 0.001 and a family wise error (FWE) correction (*p* < 0.05) was used combined with a non-stationary smoothness correction^21^.

We report partial eta squared (*η*²) as a measure of effect size.

## Results

We screened changes in the Combat Experience Scale (CES) before and after deployment. The most frequently reported events were: having experienced hostility by civilians, having seen corpses or parts of dead bodies, having seen destroyed houses and towns, having seen sick or wounded women or children, whom the soldiers could not help, and explosions in the immediate vicinity caused by improvised explosive devices (IED). In total *n* = 53 soldiers from the combat group reported increases in exposure on the death subscale and *n* = 54 on the combat operations subscale. However, in order to derive the experience during the deployment we had to subtract CES scores before and after deployment. In the total CES score 26.5% of the soldiers reported less experiences than before the deployment, which is impossible. See Limitation section for further discussion.

The distribution of male and female participant in each group did not significantly differ (Chi-Square = 0.97, *p* = 0.325).

In psychological questionnaire data, we did not observe any significant differences between the combat and control groups in changes over time for PTSD symptoms (PDS Total), state anxiety (STAI State), anxiety sensitivity (ASI), rumination (RSQ) or alcohol drinking (AUDIT) (Table 1). There was a group × time interaction for depressive symptoms (BDI-II, parametric test: *F*(1,158) = 5.25, *p* = 0.023, *η*² = 0.032; non-parametric test: *F*(1,108.33) = 63.86, *p* < 0.001), with an increase (*t*(120) = −2.60, *p* = 0.010) in the combat group and a numerical but non-significant decrease in the control group (*t*(39) = 1.15, *p* = 0.256). However, the scores at Baseline and Follow-up I were generally low (2.6–3.4) compared to previous studies on military populations and also on the general population^22^. Likewise, the numerical increase was low (0.76 points), although it reaches significance. In general, BDI values below 13 are classified as “minimal depression”.

**Fig. 1:**
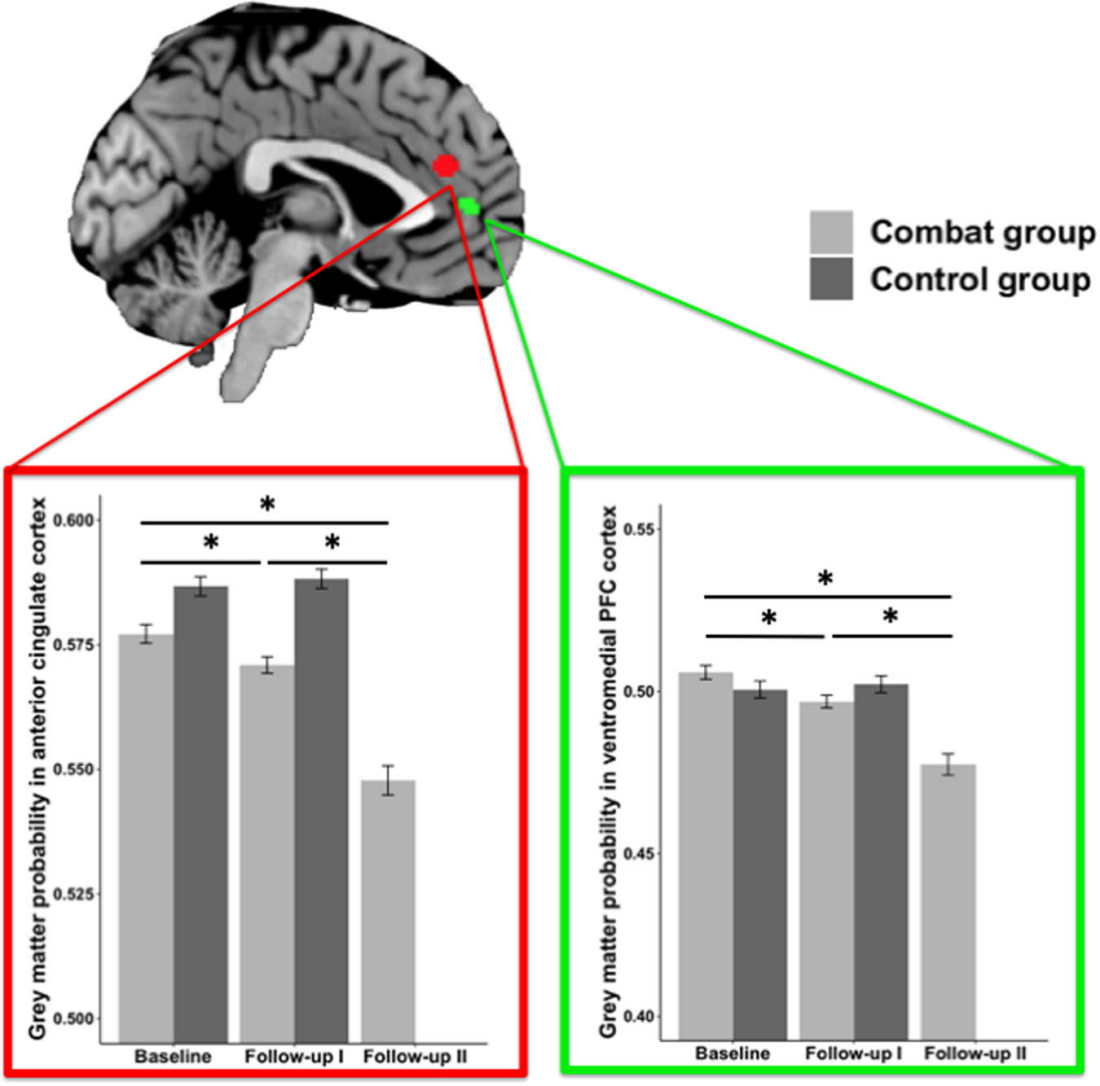
On top the two regions of interest in anterior cingulate cortex (ACC, 0, 40, 21) and ventromedial prefrontal cortex (vmPFC, 0, 49, 6) from a previous meta-analysis comparing PTSD patients and trauma-exposed controls^4^ are displayed. On the bottom illustration of the mean gray matter volume extracted from the two ROIs displayed on top. Additionally, mean values for Follow-up II about 6 months after the deployment are shown. Each asterisk indicates significant a result of a paired *t*-tests (*p* < 0.05). Within-subject error bars are computed based on Morey^39^.

Based on our a priori hypothesis we conducted ROI-analyses in brain regions from a meta-analysis comparing PTSD patients and trauma-exposed controls^4^: ACC (0,40,21), vmPFC (0,49,6) and left HC (−30,−14,−14). We found a significant group × time interaction in ACC (*F*(1,158) = 5.10, *p* = 0.025, *η*² = 0.031) and vmPFC (*F*(1,158) = 5.29, *p* = 0.023, *η*² = 0.032) (Fig. 1), but not in HC (*F*(1,158) = 1.62, *p* = 0.21). However, these effects do not survive conservative Bonferroni multiple test correction (*p* = 0.05/3 ⇒ *p* < 0.0167).

We additionally applied Bayesian statistics for the expected group × time interaction effect in the hippocampus, in order to determine evidence for the null hypothesis. Examination of the Q–Q plots suggested that the assumption of normality was not violated. A Bayesian mixed factor ANOVA revealed a Bayes Factor of 2.41 (BF01 with interaction/ BF01 without interaction: 2.689/1.115), which presents substantial evidence for the absence of an interaction.

When testing how the reductions develop over time, we observed an additional decrease from Follow-up I to Follow-up II, ~6 months after the end of deployment (*t*(91) = 8.69, *p* < 0.001), as well as between Baseline and Follow-up I (*t*(120) = 3.90, *p* < 0.001) and Baseline and Follow-up II (*t*(91) = 10.24, *p* < 0.001) for the ACC and a very similar pattern for vmPFC (Baseline to Follow-up I: *t*(120) = 4.43, *p* < 0.001, Baseline to Follow-up II: *t*(91) = 8.14, *p* < 0.001, Follow-up I to Follow-up II: *t*(91) = 4.90, *p* < 0.001). These post-hoc tests did survive multiple comparison correction. Importantly, we did not observe any significant changes in ACC (*t*(39) = −0.48, *p* = 0.634) or vmPFC (*t*(39) = −0.38, *p* = 0.709) in the control group between Baseline and Follow-up I.

Moreover, we computed a group × time interaction for left and right amygdala ROIs not finding significant effects (left amygdala: *F*(1,158) = 0.27, *p* = 0.607, right amygdala: *F*(1,158) = 1.26, *p* = 0.263).

**Fig. 2:**
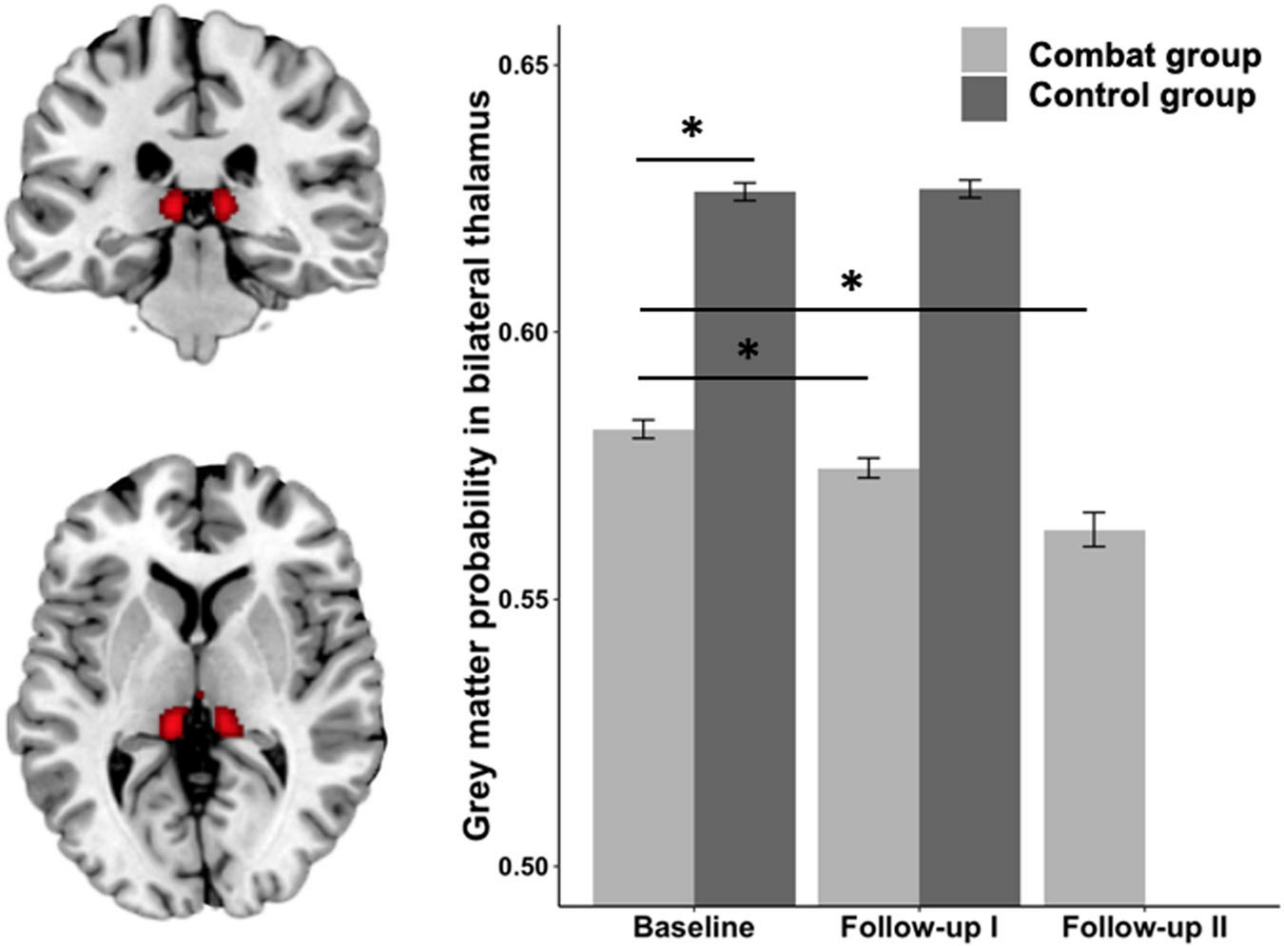
On the left results of the voxel-based morphometry whole-brain group x time interaction showing stability in control group and decrease in the combat group (FWE-corrected), on the right illustration of the mean gray matter volume extracted from the thalamus cluster. Additionally, mean values for Follow-up II about 6 months after the deployment are shown. Each asterisk indicates significant results of a paired *t*-tests (*p* < 0.05). Within-subject error bars are computed based on Morey^39^.

In the whole-brain analysis we observed one significant cluster in bilateral thalamus (MNI coordinates: −9, −30, 4, 712 voxels, FWE-corrected, Fig. 2) in the group × time interaction between Baseline and Follow-up I assessment after deployment. However, we observed a group difference between control and combat group at Baseline, with higher gray matter values in the control group (*t*(159) = −3.27, *p* = 0.001). To test how this reduction develops over time we extracted data from the same cluster at Follow-up II. We observed a significant difference between Baseline and Follow-up I (*t*(120) = 5.59, *p* < 0.001) and Baseline and Follow-up II (*t*(91) = 3.74, *p* < 0.001), but no significant additional decrease between Follow-up I and Follow-up II (*t*(91) = 1.27, *p* = 0.21). These post-hoc tests did survive multiple comparison correction. Likewise, we did not observe any significant change in bilateral thalamus (*t*(39) = −0.20, *p* = 0.844) in the control group between Baseline and Follow-up I.

When running the same analysis on white matter maps no significant results emerged.

With the goal to control for potential confounding factors we reran the analyses reported above with the additional covariates sex, TIV, education, and previous deployment status (0 or 1) finding the very same pattern of results.

In order to explore whether the observed brain changes (thalamus, ACC, vmPFC) in the combat group were related to changes in symptoms (PDS, STAI state, ASI, BDI-II, RSQ) and amount of objective combat experience (CES), we ran correlation analyses on Follow-up I–Baseline change scores. We observed no significant (*p* < 0.05) associations.

Confirming previous cross-sectional findings showing that days of deployment were negatively associated with gray matter volume in ACC and vmPFC^23^, we replicated this negative association across the entire sample for ACC (*r*(161) = −0.210, *p* = 0.008), as well as for vmPFC (*r*(161) = −0.164, *p* = 0.038) at Baseline.

## Discussion

The primary goal of this study was to investigate brain structural changes resulting from exposure to stressors during deployment. Although many soldiers in the combat group did experience combat-related stressors, we did not find any major evidence for increases in PTSD symptoms, state anxiety, anxiety sensitivity or rumination. We did observe an increase in depression scores, however, the mean BDI-II sum score was still low (3.4) even at Follow-up I and does therefore not constitute evidence for the emergence of a clinical depression. In general, the scores of the present sample are low compared to studies assessing BDI-II in the general population^22^. This might reflect fear of stigma and associated reporting biases^24^.

The absence of occurrence of PTSD in such a large population of deployed soldiers may seem surprising, but is in line with epidemiological evidence from ISAF soldiers from German military showing a 12-month *prevalence* rate for PTSD of only 2.9% in deployed soldiers and 1.2% in soldiers not deployed, and even lower 12-month *incidence* rates (deployed: 0.9% vs. not-deployed: 0.2%)^25^. A later longitudinal study on German soldiers showed no increase in PTSD rates but in depressive symptoms over the course of deployment^26^.

### Medial prefrontal cortex: ACC and vmPFC

In the brain structural analysis we focused on regions that have previously been associated with the diagnosis of PTSD^4,5^ in order to identify, to what extent alterations in these areas might be attributed to the experience of stress and trauma rather than PTSD disease. In a first analysis with ROIs taken from a meta-analysis on brain structural studies focusing on alterations in PTSD in left hippocampus, ACC and vmPFC^4^, we found evidence for a significant decrease of gray matter volume in ACC and vmPFC after compared to before deployment. Although these findings do not survive conservative Bonferroni multiple test correction, we think it is still worthwhile to interpret the finding, because we had strong a priori hypotheses for these brain regions in the fronto-medial wall, based on a previous study (in addition to the meta-analysis) in which we observed a cross-sectional correlation between lifetime days of deployment in German soldiers and gray matter decreases in ACC and vmPFC^23^. Likewise, a longitudinal study on healthy individuals scanned 3 months apart showed a negative association between the occurrence of stressful life events and ACC gray matter volume (and also HC)^27^. Animal studies have also provided evidence that exposure to chronic stress can damage the ACC of rodents^28^. Interestingly, Sekeguchi and colleagues reported a negative association between right ventral ACC before a potentially traumatic event and PTSD symptoms later on^29^. Since they only reported correlations between brain at Baseline and brain change with PTSD symptoms it is unclear, whether they would have observed ACC gray matter decline over time. However, it is remarkable that the same brain region has been implicated before and in fact been shown to be related to PTSD symptoms in a sample, in which symptom levels were also overall low.

What surprised us was the absence of recovery but instead an additional decrease in ACC and vmPFC gray matter between Follow-up I and Follow-up II, ~6 months after the end of deployment. The fact that the control group was not re-invited to Follow-up II prevents a formal group x time interaction analysis. However, we did not observe significant changes in the control group between Baseline and Follow-up I, which were ~7 months apart, so that it is unlikely that the change observed in deployed soldiers between Follow-up I and II can be attributed to natural aging processes. Previous studies on the effects of stress on brain structure did not assess the timescale of the observed changes. In the prior cross-sectional study associating days of deployment to decreases in ACC, additional volume reductions occurring after deployment would have gone unnoticed and would have simply contributed to the overall effect^23^. The study by Papagni and colleagues^27^ assessed participants with a 3-months interval, and associated brain structure with stressful life events but did not include a later follow-up that could have revealed these delayed alterations. The observation that the volume decrease was not limited to the period of actual stress but continued much later is highly relevant in terms of the timepoint at which consequences of deployment can and should be assessed and opens new perspectives to early interventions, once soldiers return from deployment that may help to prevent further decline in the ACC and vmPFC after deployment. These post-deployment decrements in brain structure may hint at the initiation of a pathobiological process, seemingly below a symptom threshold, that may potentially be involved in the pathogenetic processes linked to depression and pave the way to future mental health problems. In order to investigate this in more depth, it would be useful if future studies would include even longer delays until follow-up, to estimate whether there is any sign of recovery at a later stage.

Functionally, the vmPFC as well as the rostral ACC has been implicated in extinction learning^30^, which may explain part of the PTSD symptoms. Moreover, the ACC and vmPFC have also been highlighted in their role in contextual processing during which organisms disambiguate cues and derive situation-specific meaning from the world^31^.

### Hippocampus and amygdala

We did not observe any significant changes in the HC, neither in our ROI analysis in an a priori cluster in left HC^4^, nor in the whole-brain analysis. This absence of change fits to the twin study by Gilbertson, demonstrating that lower HC volume most likely constitutes a risk factor for PTSD that is shared by twins^8^.

Since amygdala structure has also been implicated in PTSD^32^ and alterations of functional activity in amygdala have been demonstrated in the context of combat exposure^11^, we conducted a ROI analysis in bilateral amygdala. However, we did not observe any significant structural changes over time.

### Thalamus

In the whole-brain analysis that we conducted without any a priori focus on specific brain regions, we found a significant reduction in bilateral medial pulvinar thalamic nucleus in the deployed soldiers in comparison to soldiers who stayed home. However, this reduction did not progress after the end of deployment. Up to now, the thalamus is not very prominently discussed in studies focusing on brain structural alterations in PTSD or stress exposure. Recently, an animal study has been published, in which rats were experimentally exposed to stress and later scanned using MRI and VBM methodology. Rodents were exposed to three stressors: 2 h restraint, 20 min group swim, and exposure to ether until loss of consciousness, which is a pre-clinical model for PTSD. In line with our present findings, the authors report volume decreases in thalamus and visual cortex, but not in medial prefrontal or hippocampal regions^33^. Although this is one of the few empirical studies linking thalamus to stress and fear, the thalamus has been described as part of a context processing hippocampal-prefrontal-thalamic circuitry. Liberzon and colleagues describe this network for contextualization to explain PTSD pathophysiology when it becomes dysregulated^31^. As described in the context of fear conditioning, perceptual input is relayed to the thalamus and then to the amygdala and to effector systems^34–36^.

In a similar study by van Wingen and colleagues^13^, comparing brain measures before and after deployment in soldiers to a control group, white matter alterations in bilateral midbrain were detected, which adjoins the thalamus. In contrast to the present results, the authors did observe recovery after 1.5 years. Although we observed no further decline in thalamus between Follow-up I and II, we still saw a significant decline comparing Baseline to Follow-up II. This could be due to the fact that our second follow-up was earlier, namely about 6.5 months after return, or reflect differences between the two brain regions. The fact that van Wingen and colleagues^11^ observed no gray matter changes could be due to the considerably smaller sample size. However, Sekiguchi and colleagues^29,37^ (*n* = 42/30 survivors) did report whole-brain changes in left orbitofrontal cortex gray matter in earthquake survivors to be associated with PTSD symptoms and observed gray matter decreases in hippocampus and medial as well as orbitofrontal cortex between pre, post and 1 year after an earthquake experience (*n* = 37)^14^.

### Association with psychological changes or combat experiences

We did not find any associations between brain changes and changes in symptoms or combat experiences. This may seem surprising, since the previous study on brain effects of combat stress did show a covariation of amygdala connectivity with perceived threat^11^. Since the present study did not include questions on perceived threat, we could not directly test this association. However, in line with previous studies we did find a negative association between days of deployment soldiers reported and gray matter volume in ACC and vmPFC, paralleling our previously reported cross-sectional data in soldiers of the German military^23^.

### Limitations

An obvious but unavoidable problem of the study design is the lack of random assignment. For most of the soldiers the deployment was scheduled before entry into the study and most had already undergone preparation for the deployment. Overruling these procedures would neither have been possible nor ethically justifiable. Future studies may want to recruit the same number of control participants as the ones in the combat exposure group, and may want to avoid variability in the time between post-test and follow-up assessment. Another problem is, that we cannot disentangle the effects of exposure to traumatic experiences from other situational differences, that deployed soldiers were facing such as separation from family and friends, different diet, temperature and changes in habits such as exercise.

Another limitation were participants’ concerns about potential implications of questionnaire responses, which may have resulted in underreporting of symptoms. The stigma of mental health problems in the military is widely acknowledged^38^ and since disclosing mental illness can lead to gossip, discrimination as well as negative career consequences^24^, soldiers may have underreported symptoms. However, we took precautions by reminding participants repeatedly that the data is collected for research purposes and analyzed on group-level only. Nevertheless, the present population did not seem to have developed relevant PTSD or depressive symptoms, which should caution the clinical interpretation of the results presented. Future studies are needed to investigate the reliability of the combat experience assessment by means of CES since in the present study more than a quarter of the participants in the combat group reported less lifetime experiences after compared to before the deployment. Moreover, we would recommend that future studies attempt to assess the subjective stress elicited by the mere fact of being deployed into a war zone above and beyond particular combat experiences.

## Conclusion

The present findings reveal that deployment to regions of war leads to volume reductions in ACC, vmPFC, and thalamus in soldiers compared with non-deployed soldiers. Most interestingly, this decrease in brain volume did not only occur during the period of deployment, but continued over the following 6 months after deployment in ACC and vmPFC. This new finding may have implications for the timing of the assessment of symptoms after occurrence of stressful life events and may point at a window of opportunity for early interventions that may help prevent further neural decline after the combat or related long-term stress exposure. Moreover, these post-deployment decrements may hint at the initiation of a pathobiological process that may potentially pave the way to future mental health problems. Based on the results of the present study, we conclude that alterations in medial prefrontal cortex are rather the consequence of stress/trauma exposure rather than a vulnerability factor for PTSD.
